# Impact of Prolonged Fasting on the Oral Microbiome in Patients With Metabolic Syndrome: An Exploratory Secondary Analysis

**DOI:** 10.1111/jcpe.14171

**Published:** 2025-05-12

**Authors:** C. L. Pappe, S. Kleine Bardenhorst, K. Prior, N. Steckhan, A. Michalsen, B. Ehmke, H. Dommisch, D. Hagenfeld

**Affiliations:** ^1^ Department of Periodontology, Charité – Universitätsmedizin Berlin, Corporate Member of Freie Universität Berlin and Humboldt‐Universität zu Berlin Oral Medicine and Oral Surgery Berlin Germany; ^2^ Department of Periodontology and Operative Dentistry University Hospital of Münster Münster Germany; ^3^ Digital Health‐Connected Healthcare, Hasso Plattner Institute University of Potsdam Potsdam Germany; ^4^ Evidence‐Based Digital Diabetology, Medical Faculty Carl Gustav Carus, Department of Medicine III, Prevention and Care of Type 2 Diabetes Technical University of Dresden Dresden Germany; ^5^ Institute of Social Medicine, Epidemiology and Health Economics, Charité–Universitätsmedizin Berlin Corporate Member of Freie Universität Berlin and Humboldt‐Universität zu Berlin Berlin Germany; ^6^ Department of Internal and Integrative Medicine Immanuel Hospital Berlin Berlin Germany

**Keywords:** bleeding on probing, fasting, gingival crevicular fluid, microbiota, periodontitis

## Abstract

**Aim:**

To evaluate the effect of prolonged fasting on the oral microbiome in patients with metabolic syndrome (MetS).

**Materials and Methods:**

This follow‐up study evaluated changes in the oral microbiome in a sub‐cohort of 42 patients with MetS during prolonged fasting. Periodontal parameters such as bleeding on probing (BOP), plaque index (PI) and gingival crevicular fluid (GCF) measured in Periotron units (PU) as well as supra‐ and subgingival plaque samples were taken at baseline (T1), after 5–10 days of prolonged fasting (T2) and at 4–5‐month follow‐up (T3). Sequencing of the V4 hypervariable region of the 16S rRNA gene was performed to analyse the microbiome composition.

**Results:**

Significant reductions were observed in BOP: 36.4% ± 18.2% to 30.4% ± 15.6% (*p* = 0.01), PI: 66.9% ± 19.5% to 58.8% ± 23.4% (*p* < 0.01) and GCF: 83.6 ± 27.8 PU to 67.9 ± 30.3 PU (*p* < 0.01) post fasting. Microbiome α‐ and β‐diversity did not change significantly. However, significant changes in specific bacterial genera were noted: *Lachnospiraceae* [G‐3] increased only in subgingival samples; *Eikenella* and *Peptostreptococcaceae* [XI][G‐7] increased; while *Mitsuokella* and *Atopobium* decreased in both sub‐ and supra‐gingival samples.

**Conclusion:**

Within the constraints of this analysis, prolonged fasting was found to be associated with reduced periodontal inflammation and selected shifts in the oral microbial composition. Larger controlled trials are needed to confirm these exploratory findings and determine their clinical relevance.

## 1 Introduction

The oral microbiome plays a critical role in the development of oral inflammatory diseases. Its presence is vital for immune response regulation, as evidenced by studies on germ‐free mice and experimental gingivitis models (Löe et al. 1965; Rovin et al. 1966). A healthy periodontal state maintains a balanced relationship between the immune system and the oral microbiome (Meyle and Chapple 2015; Ximénez‐Fyvie et al. 2000). However, inflammatory processes can disrupt this balance, fostering an environment conducive to inflammophilic pathogens (Marsh and Devine 2011). Periodontitis is characterised by an increase in anaerobic gram‐negative bacteria, leading to an excessive and destructive immune response (Hajishengallis et al. 2011; Socransky et al. 1998). Thus, periodontal therapy involves regular disruption of supra‐ and subgingival biofilms (Axelsson et al. 2004; Löe et al. 1965; Trombelli et al. 2015). If applied continuously, this maintenance therapy can result in preventing periodontal breakdown (Sanz et al. 2020). Various lifestyle factors, including a Western diet, stress and tobacco use, can foster repeated periodontal inflammation and lead to further bone loss (Nyvad and Takahashi 2020; Sedghi et al. 2021).

Thus, focusing on diet has become more and more important in recent years. High‐sucrose diets, for instance, promote cariogenic streptococci, causing lower salivary pH and therefore caries (Anderson et al. 2018, 2020). Different dietary patterns can potentially influence the subgingival environment, as seen in human evolution (Lassalle et al. 2018). Short‐term dietary interventions have shown anti‐inflammatory effects on periodontal parameters in both experimental gingivitis models and untreated periodontal disease cohorts (Bartha et al. 2022; Jenzsch et al. 2009; Woelber et al. 2016, 2019). However, changes in the oral microbiome during these interventions are heterogeneous. For example, nitrate‐rich diets have the potential to affect the oral microbiome (Jockel‐Schneider et al. 2021; Rosier et al. 2020; Rosier et al. 2024), whereas other studies found no significant subgingival changes after oral‐health‐optimised diets (Baumgartner et al. 2009; Woelber et al. 2019). The impact of fasting‐induced total food deprivation on the oral microbiome remains insufficiently explored. Further research could enhance our understanding of the interplay between diet, the microbiome and inflammation.

Fasting, the voluntary abstinence from some or all foods, confers anti‐inflammatory and metabolic benefits (Koppold et al. 2024). Mechanisms include reductions in pro‐inflammatory markers IGF‐1 and glucose levels, alongside increased stress resistance, which may potentially affect the periodontium (Parveen 2021; Parveen and Alhazmi 2022). A previous study by our group indicated that a 5–10‐day multimodal fasting intervention significantly reduced periodontal inflammation in women with metabolic syndrome (MetS) (Pappe et al. 2021). A recent study on intermittent fasting found reductions in bleeding on probing (BOP) and periodontal probing depth (PPD) in 14 patients with increased body mass index (BMI) after 6 months. However, no changes in subgingival α‐ or β‐diversity were observed. When analysing only patients with ≥ 60% PPD reduction, significant changes in several taxa were identified (Lira‐Junior et al. 2024). Studies on prolonged fasting have shown significant shifts in the gut microbiome post fasting in both healthy and diseased patients (Maifeld et al. 2021; Mesnage et al. 2019), along with alterations in several salivary taxa (Loumé et al. 2024). To the best of our knowledge, there is no study investigating the effects of prolonged fasting on the subgingival oral microbiome. We hypothesised that a prolonged fasting intervention of 5–10 days will alter the composition of the supra‐ and sub‐gingival oral microbiome, thereby disadvantaging inflammatory species.

## 2 Materials and Methods

### 2.1 Study Design, Population and Ethics

This analysis included 42 patients from the original cohort from Pappe et al. (2021), with the difference that we included both men and women together. The cohort comprised all patients initially diagnosed with MetS. Included were non‐smoking patients aged between 18 and 80 years with a diagnosis of MetS who planned to fast, and they were enrolled after signing informed consent.

The exclusion criteria were as follows: general medical conditions that do not allow fasting therapy; patients with a history of endocarditis; intake of antibiotics (minimum of 4 months earlier); alcohol abuse; drug abuse; psychological disorders; eating disorders; dementia; pregnancy; and breast feeding mothers.

A comprehensive description of the study design, intervention, eligibility, blinding and calibration can be found elsewhere (Pappe et al. 2021).

Clinical parameters and samples were taken at baseline (T1), after the fasting intervention (T2) and at 4–5‐month follow‐up (T3). Deviating from the previous publication, both male and female patients were analysed together.

Patients attended a 2‐week‐long fasting programme in an integrative ward (Immanuel Clinic Berlin, Wannsee, Germany) based on Buchinger's criteria (Buchinger 1979; Li et al. 2013), with daily energy intake restricted to 300–500 kcal (1256–2092 kJ). The fasting regimen included consumption of vegetable broths, fruit juices and unlimited water or tea (but excluding acidic fruit tea). Patients were provided with mineral supplements to ensure adequate nutrient intake. Fasting lasted 4–10 days depending on individual health and well‐being, followed by a reduced‐calorie diet (1000–1300 kcal/day) (for more details see Pappe et al. 2021). This prospective clinical cohort study received approval from the institutional ethical review board of Charité—Universitätsmedizin Berlin (EA4/054/15) and followed the Declaration of Helsinki (1975, 2013). Patients provided signed informed consent before fasting and the dental examination.

### 2.2 Clinical Data Collection

Clinical parameters, such as periodontal screening index (PSI), full‐mouth BOP, gingival bleeding index (GBI), plaque index (PI) and gingival crevicular fluid (GCF) volume, were assessed at all timepoints (T1–T3) using standardised protocols (Pappe et al. 2021). Periodontal parameters were measured using a pressure‐sensitive periodontal probe (0.25 N; Kerr; Hawe Click‐Probe 3/6/9/12, 3/Art. No. 1390, Kerr GmbH, Biberach, Germany) by one experienced periodontist (C.L.P.) to ensure consistency (Pappe et al. 2021). Blood samples for C‐reactive protein (CRP), fasting glucose levels (FGLU) and glycated haemoglobin (HbA1c) were collected at T1–T3 and analysed in a certified laboratory (MVZ Laborverbund GmbH, Henningsdorf, Germany). The primary outcome in the original cohort was BOP (T1–T2).

### 2.3 Collection of Microbiological Samples

Microbiological samples were collected under dried conditions using cotton rolls from the area of the deepest periodontal pocket of each patient at all timepoints. Supragingival samples were taken first, followed by subgingival samples, each with a sterile curette. The specimens were preserved in sterile 1.5‐mL safe‐lock tubes at –80°C until further processing.

### 2.4 16S rRNA Gene Sequencing

Cells from oral samples were first collected and washed by centrifugation at 5000*g* for 5 min, followed by re‐suspension in ice‐cold PBS, a second centrifugation and supernatant removal. The cells were then lysed by re‐suspending them in a digestion buffer (100 mM NaCl, 10 mM TrisCl at pH 8, 25 mM EDTA at pH 8, 0.5% SDS, 0.1 mg/mL proteinase K), proportionate to the cell count, and incubating at 56°C for 1 h with shaking. Nucleic acids were extracted using an equal volume of phenol/chloroform/isoamyl alcohol, followed by centrifugation at 17,000*g* for 10 min. The aqueous top layer was transferred to a new tube, to which ammonium acetate and ethanol were added, and the mixture was centrifuged at 17,000*g* for 2 min to precipitate the DNA. The DNA pellet was rinsed with 70% ethanol, air‐dried and re‐suspended in TE buffer, and then dissolved at room temperature or 65°C. The purified DNA was stored at 4°C for future applications. Paired‐end V4 16S rRNA gene libraries were prepared, normalised, pooled and sequenced using an Illumina MiSeq system according to the methods described earlier (Hagenfeld et al. 2023).

Bioinformatics analysis involved initial processing of the FastQ files using Illumina's MiSeq Control Software v.2.6.2.1 and Cutadapt v.4.4 (Martin 2011) for adapter and primer removal. Further analysis was conducted using the DADA2 package v.1.26.0 (Callahan et al. 2016) in R v.4.2.2 (R Development Core Team 2017), including trimming of low‐quality bases and filtering of low‐quality reads, as described by Hagenfeld et al. (2023). Primer‐trimmed reads were submitted to the European Nucleotide Archive (http://www.ebi.ac.uk/ena/) of EMBL European Bioinformatics Institute under the study accession number PRJEB89223.

Genus‐ and species‐level taxonomic classification of ribosomal sequence variants (RSVs) was performed using a Bayesian classifier with the eHOMD v.15.23 training set (Chen et al. 2010). A table with RSV sequences, eHOMD‐, as well as SILVA‐ (v. 138.1) taxonomy was created to allow for reproducibility (Pruesse et al. 2007) (Table S1). To ensure data quality, a minimum abundance filter of 100 was applied to remove rare sequence variants.

### 2.5 Missing Data

Initially, 45 patients were enrolled in the study, but missing data at different timepoints led to a reduced dataset. During fasting, one patient withdrew because of antibiotic use and another for personal reasons (*n* = 2). Additionally, plaque samples were missing at T2 (*n* = 1) and T3 (*n* = 23). This resulted in 43 patients with complete samples at baseline, 42 who provided samples post fasting, and only 21 available for follow‐up. Given these numbers, comprehensive statistical analyses were feasible between T1 and T2, with a power of 80% to detect a conservative effect size of 0.4. However, the power dropped to 53% with only 21 observations for T1/T3 comparisons, necessitating a shift to descriptive sensitivity analysis for the follow‐up phase due to the substantially reduced sample size and limited statistical power.

### 2.6 Statistical Analysis

Statistical analyses were performed using R v.4.2.2. Continuous variables were expressed as means ± standard deviations, and categorical variables were presented as frequencies and percentages. Comparisons between baseline (T1) and post‐fasting (T2) measurements were made using Wilcoxon signed‐rank tests for non‐normally‐distributed data.

Multivariable analyses were conducted to adjust for potential confounders, including age, sex and baseline microbiome status. Mixed‐effect modelling was employed to assess whether the diversity of the microbiome changes after the fasting regimen, accounting for the variable fasting durations. The analysis included the interaction term with the timepoint to assess the effect of fasting duration, with the effect on baseline microbiome set to zero. The random part of the model accounted for multiple samples from the same patients. α‐Diversity indices (richness, Shannon, Simpson) and β‐diversity metrics (Bray–Curtis dissimilarity) were calculated to evaluate microbiome diversity. The genus‐level subgingival dysbiosis index (SMDI) was calculated to determine if fasting impacts sub‐gingival dysbiosis (Chen et al. 2022). Principal coordinates analysis (PCoA) based on Bray–Curtis dissimilarity was performed to visualise microbiome composition changes over time. Differences in microbial community structure were assessed using PERMANOVA to detect group differences, and distance‐based redundancy analysis (dbRDA) was used to detect linear associations with host factors.

To identify differentially abundant taxa, we used three complementary approaches: ANCOM‐BC, LinDA and Wilcoxon tests. ANCOM‐BC (analysis of composition of microbiomes with bias correction) (Lin and Peddada 2020) and LinDA (linear regression framework for differential abundance analysis) (Zhou et al. 2022) were used to detect differentially abundant taxa while accounting for multiple covariates and adjusting for potential sequencing biases using log‐ratio transformations. Wilcoxon signed‐rank tests were performed using relative abundances for paired unadjusted comparisons to detect differentially abundant taxa between baseline and post‐fasting samples. *p*‐values were corrected for multiple testing, and an adjusted *p*‐value < 0.05 was considered statistically significant. All differential abundance analyses were performed at the genus and species level; however, species‐level results only were summarised in the sensitivity analysis because of limited species‐level resolution of the short reads of the V4 hypervariable region of the 16S rRNA‐gene.

## 3 Results

The study included a total of 43 patients diagnosed with MetS between June and November 2016. The mean age of the patients was 64.02 ± 7.61 years. The cohort comprised 7 males (16%) and 36 females (84%). The mean HbA1c level at baseline was 6.17% ± 1.32%. All patients underwent a multimodal prolonged fasting intervention with varying durations. Specifically, 1 patient (2.3%) fasted for 5 days, 6 patients (14%) for 6 days, 7 patients (16%) for 7 days, 12 patients (28%) for 8 days, 6 patients (14%) for 9 days and 11 patients (26%) for 10 days.

Periodontal status at baseline was assessed using the PSI. The majority of patients (72%) had at least one PSI code of 4, indicating suspected severe periodontal disease, while 11 patients (26%) had a PSI score of two times 3, leading also to suspected periodontal disease. One patient (2.3%) had a maximum PSI score of 2. Regarding smoking status, 11 patients (26%) had quit smoking for more than 2 years, and 32 patients (74%) were lifelong non‐smokers.

For the analysed population, sociodemographic data, anthropometric data and periodontal status at baseline are summarised in Table 1.

**TABLE 1:** Participant characteristics at baseline (T1).

Baseline characteristic		Fasting group (*n* = 43)
Age (years)	Mean (SD)	64.02 (7.61)
Sex		
Male	*n* (%)	7 (16%)
Female	*n* (%)	36 (84%)
HbA1c (%)	Mean (SD)	6.17 (1.32)^a^
FGLU (mg/dL)	Mean (SD)	115 (40)
BMI	Mean (SD)	35.9 (7.14)
Fasting duration		
5 days	*n* (%)	1 (2.3%)
6 days	*n* (%)	6 (14%)
7 days	*n* (%)	7 (16%)
8 days	*n* (%)	12 (28%)
9 days	*n* (%)	6 (14%)
10 days	*n* (%)	11 (26%)
PSI		
2	*n* (%)	1 (2.3%)
3	*n* (%)	11 (26%)
4	*n* (%)	31 (72%)
Smoking status		
> 2 years non‐smoker	*n* (%)	11 (26%)
Lifelong non‐smoker	*n* (%)	32 (74%)

Abbreviations: BMI, body mass index; FGLU, fasting glucose; HbA1c, percentage of glycated haemoglobin; *n*, number of patients; PSI, periodontal screening index (min. 2 times code 3, min. once code 4); SD, standard deviation.

^a^ One missing (*n* = 42).

### 3.1 Changes in Clinical Parameters

Following the fasting intervention, significant changes were observed in several clinical parameters. The primary outcome parameter (i.e., BOP) showed a significant reduction from 36.4% ± 18.2% at baseline (T1) to 30.4% ± 15.6% post fasting (T2) (*p* = 0.01). Similarly, GBI decreased significantly from 36.6% ± 19.6% at T1 to 29.7% ± 21.1% at T2 (*p* = 0.02).

PI also showed a notable reduction, decreasing from 66.9% ± 19.5% at T1 to 58.8% ± 23.4% at T2 (*p* < 0.01). Additionally, GCF significantly decreased from 83.6 ± 27.8 PU at T1 to 67.9 ± 30.3 PU at T2 (*p* < 0.01), indicating a reduction in inflammation. The pH of the oral environment showed a significant decrease from 6.96 ± 0.499 at T1 to 6.35 ± 0.525 at T2 (*p* < 0.01), suggesting an alteration in the oral biochemical environment post fasting. However, CRP levels did not show any significant change, increasing from 6.00 ± 6.20 mg/L at T1 to 7.58 ± 8.65 mg/L at T2 (*p* = 0.16).

FGLU levels decreased from 115 ± 46.0 mg/dL at T1 to 84.0 ± 52.5 mg/dL at T2 (*p* < 0.01) (Table 2).

**TABLE 2:** Clinical parameters at baseline (T1) and post fasting (T2).

Clinical parameter	*n*	Fasting group		
		T1	T2	T1 − T2
		Baseline (mean ± SD)	Post fasting (mean ± SD)	Intra‐*p*‐value^a^
BOP (%)	27	36.4 ± 18.2	30.4 ± 15.6	**0.01**
GBI (%)	16	36.6 ± 19.6	29.7 ± 21.1	**0.02**
PI (%)	43	66.9 ± 19.5	58.8 ± 23.4	**< 0.01**
GCF (PU)	43	83.6 ± 27.8	67.9 ± 30.3	**< 0.01**
pH	43	6.96 ± 0.499	6.35 ± 0.525	**< 0.01**
CRP (mg/L)	41	6.00 ± 6.20	7.58 ± 8.65	0.16
FGLU (mg/dL)	43	115.0 ± 46.0	84.0 ± 52.5	**< 0.01**
BMI (kg/m^2^)	43	35.9 ± 7.14	34.3 ± 6.83	**< 0.01**

*Note*: Entries in bold indicate statistical significance *p* < 0.05.

Abbreviations: BMI, body mass index; BOP, bleeding on probing; CRP, c‐reactive protein; FGLU, fasting glucose; GBI, gingival bleeding index; GCF, gingival crevicular fluid; PI, plaque index; SD, standard deviation.

^a^ Wilcoxon signed‐rank test.

### 3.2 Microbiome Composition and Diversity

SMDI and α‐diversity, including richness, Shannon index and Simpson Index, were controlled for age, differences in fasting duration and sequencing depth to evaluate changes in the microbiome composition from T1 to T2. The results showed no statistically significant differences in any of the α‐diversity measures between the two timepoints for both subgingival and supragingival samples. Non‐significant mean differences for each index are illustrated in Figure S1. For subgingival samples, the richness at T1 had a mean value slightly lower than at T2 (model estimate [E]: 7.14 ± 6.13 standard‐error [SE]; adjusted *p*‐value [*p*.adj.]: 0.29). Similarly, supragingival samples showed a mean richness slightly increasing from T1 to T2, but the differences were not statistically significant (E: 4.96 ± 6.93 SE; *p*.adj.: 0.48). The Shannon diversity index showed minimal changes between T1 and T2 for both subgingival (E: 0.46 ± 0.20 SE; *p*.adj.: 0.13) and supragingival (E: 0.36 ± 0.21 SE; *p*.adj.: 0.18) samples. The mean values remained nearly constant, and no shifts were detected. The Simpson diversity index also showed no changes for both subgingival (E: 4.91 ± 2.58 SE; *p*.adj.: 0.15) and supragingival (E: 5.06 ± 2.32 SE; *p*.adj.: 0.13) samples between the two timepoints. SMDI did not change statistically significantly in the subgingival samples (E: 1.19 ± 0.88 SE; *p*.adj.: 0.26).

β‐Diversity was assessed to evaluate changes in microbial community structure between baseline (T1) and post fasting (T2). PCoA based on Bray–Curtis dissimilarity was used to visualise the differences in microbiome composition at the two timepoints. Figure S2 presents PCoA plots for both supragingival and subgingival samples. Each point represents the microbial community of an individual sample, with blue points indicating T1 and orange points indicating T2.

In the PCoA plot for supragingival samples, partial separation between T1 and T2 is observed along the first principal coordinate (PC1), which explains 35.41% of the total variation. The second principal coordinate (PC2) explains 23.56% of the variation. The ellipses representing the 95% confidence intervals for T1 and T2 show some overlap, indicating moderate changes in the microbial community structure post fasting. Similarly, the PCoA plot for subgingival samples shows a degree of separation between T1 and T2 along PC1 (36.09%) and PC2 (20.24%), with overlapping 95% confidence intervals, suggesting changes in the microbial composition, but not a complete shift (Figure S2).

A PERMANOVA analysis, controlled for baseline microbiome composition, different fasting durations and age, was conducted to statistically assess the differences in microbial community structure between the timepoints. The results of this analysis indicated that there were no differences in β‐diversity between T1 and T2 for both supragingival and subgingival samples. However, β‐diversity was associated with age (*p* = 0.03) in supragingival samples, a finding further confirmed by dbRDA analysis.

### 3.3 Differential Abundance Analysis

**FIGURE 1:**
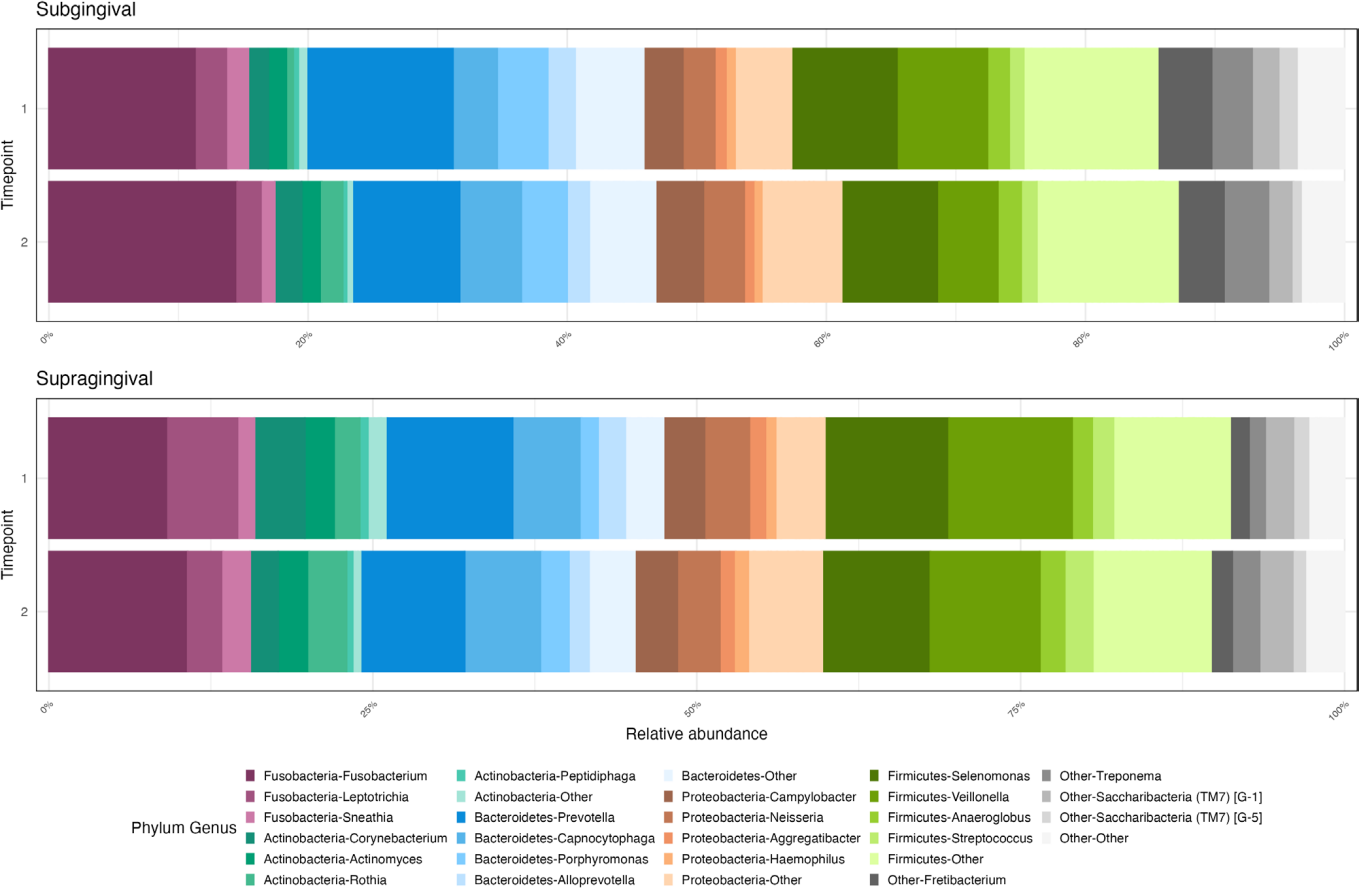
Stacked bar charts showing the relative abundance of bacterial phyla and genera in subgingival (top) and supragingival (bottom) plaque samples before (T1) and after (T2) the fasting intervention. Each colour represents a distinct taxonomic group.

Before the fasting intervention (T1), the microbial profiles of both subgingival and supragingival samples were predominantly characterised by the phyla *Firmicutes*, *Bacteroidetes*, *Fusobacteria* and *Proteobacteria*. At the genus level, *Selenomonas*, *Prevotella* and *Fusobacterium* were notably abundant. Following the fasting intervention (T2), the overall relative abundances of these major phyla and genera remained largely stable, with only minor fluctuations observed (Figure 1).

**FIGURE 2:**
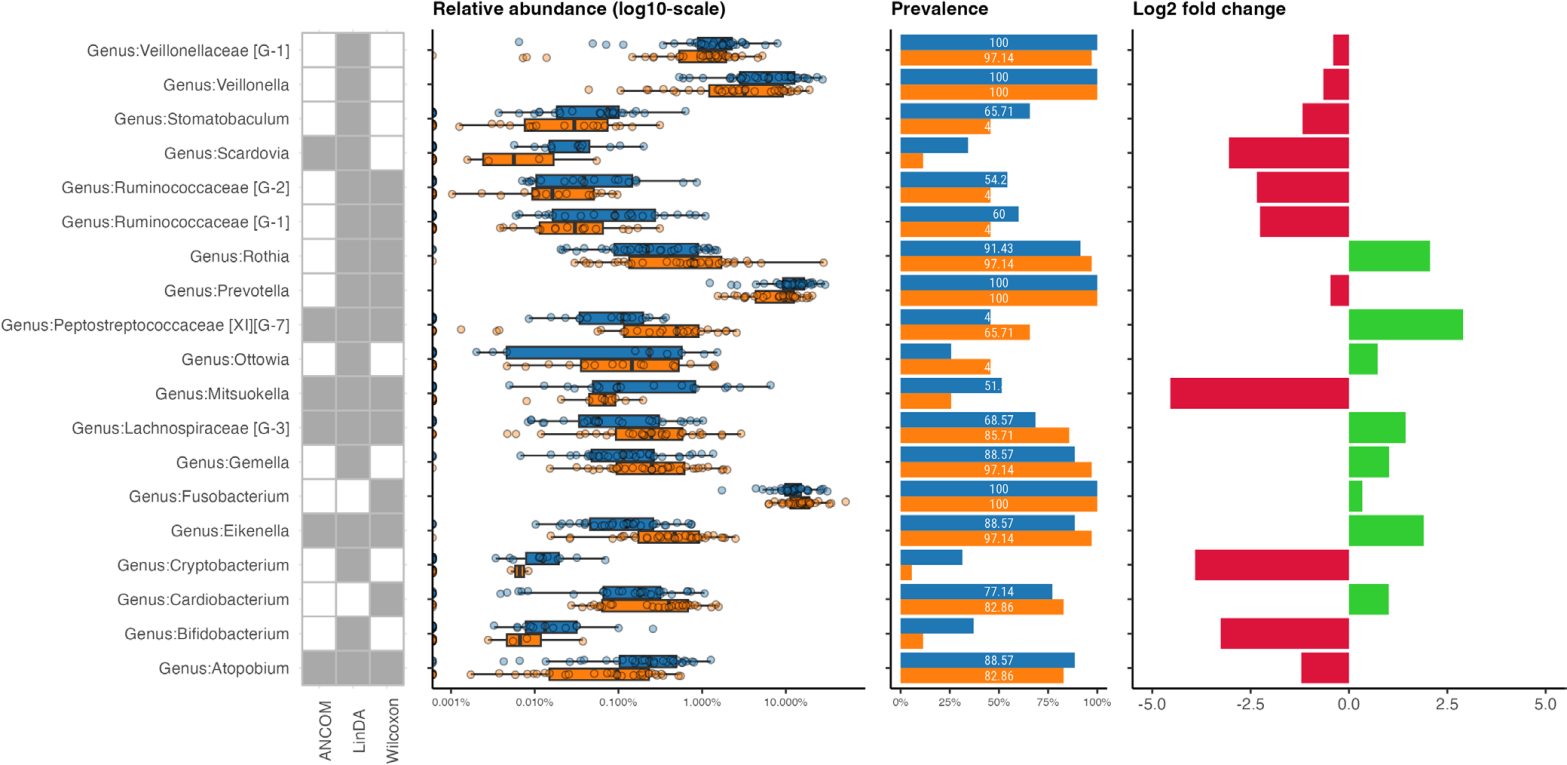
Differential abundance summary for subgingival samples, showing the relative abundance (log_10_ scale), prevalence and log_2_‐fold change of genus‐level taxa significantly different between baseline (blue) and post fasting (orange). Genera are marked green when they show significantly different abundances in the respective models (ANCOM, LinDA and Wilcoxon).

**FIGURE 3:**
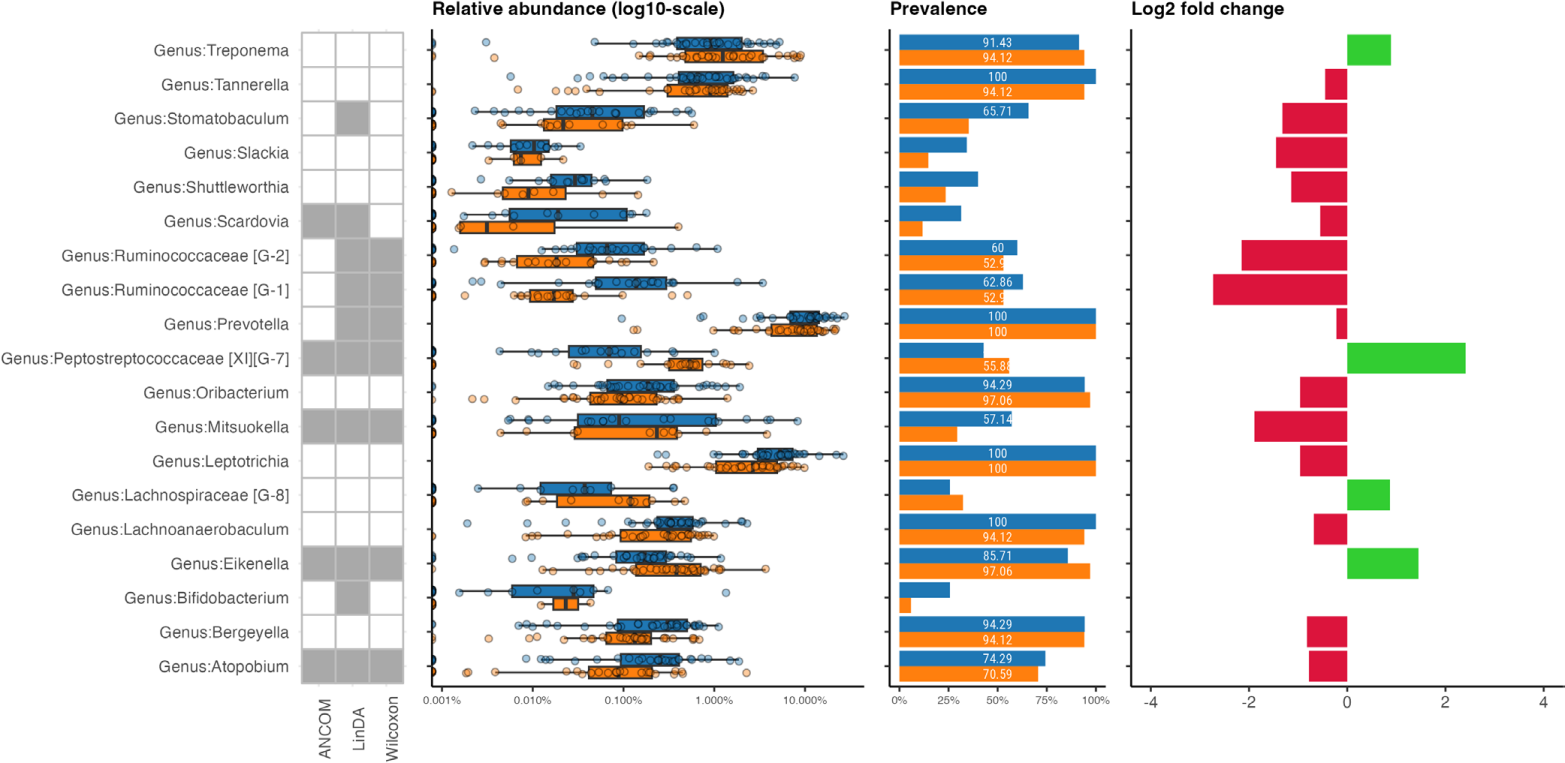
Differential abundance summary for supragingival samples, showing the relative abundance (log_10_ scale), prevalence, and log_2_‐fold change of genus‐level taxa significantly different between baseline (blue) and post fasting (orange). Genera are marked green when they show significantly different abundances in the respective models (ANCOM, LinDA and Wilcoxon).

Five genera from subgingival samples (Figure 2) and four genera from supragingival samples (Figure 3) showed consistent changes in relative abundance post fasting, as highlighted by their significant identification in all three models (ANCOM‐BC, LinDA and Wilcoxon). In subgingival samples, genera *Mitsuokella* and *Atopobium* showed significant decreases in relative abundance from T1 to T2. Conversely, genera *Peptostreptococcaceae* [XI][G‐7], *Lachnospiraceae* [G‐3] and *Eikenella* showed significant increases in relative abundance post fasting. Except for *Lachnospiraceae* [G‐3], which showed no differential abundance in the supragingival samples, *Peptostreptococcaceae* [XI][G‐7], *Mitsuokella*, *Eikenella* and *Atopobium* also showed robustly significant different abundances in the supragingival samples in all three differential abundance models.

*Mitsuokella* and *Atopobium* decreased in both sub‐ and supragingival samples. In the subgingival samples, the log_2_‐fold change indicates notable shifts, with *Mitsuokella* decreased by up to 2–3‐fold, while *Atopobium* decreased by a lower magnitude below one‐fold. The genus *Peptostreptococcaceae* [XI][G‐7] increased above two‐fold in the sub‐ and supra‐gingival samples. The other significantly different abundant genera in the subgingival samples showed only minor shifts of around 1‐fold. Prevalence data indicate that while some genera, such as *Eikenella* and *Atopobium*, were consistently present in most subgingival samples at both timepoints, others like the genera *Peptostreptococcaceae* [XI][G‐7], *Mitsuokella* and *Lachnospiraceae* [G‐3] exhibited marked changes in prevalence between T1 and T2.

Overall, the analysis reveals significant shifts in bacterial genera post fasting, with only a few genera showing robust differential abundance. This suggests that fasting may influence only specific bacterial genera rather than the entire microbial communities in the subgingival environment.

### 3.4 Sensitivity Analysis

In the species‐level differential abundance analysis, *Eikenella corrodens* increased in both subgingival (Figure S3) and supragingival samples (Figure S4), while Lachnospiraceae [G3] with unknown species increased only in the subgingival samples directly post fasting (T2).

Only 21 of the 43 patients returned for a follow‐up visit after 4–5 months (T3). While a trend towards a stronger reduction in α‐diversity was noted, none of the changes in α‐ or β‐diversity was statistically significant. Additionally, no bacterial genera showed statistically significant differential abundance between T1 and T3.

## 4 Discussion

This study investigated the impact of prolonged multimodal fasting on the subgingival and supragingival oral microbiome in patients with MetS. Significant reductions in clinical parameters were observed post fasting, accompanied by specific changes in bacterial genera. However, no changes were observed in microbiome diversity measures directly post fasting and at the follow‐up visit after 4–5 months.

The study population consisted predominantly of older adults with MetS, which is relevant because both age and metabolic status can influence periodontal health and microbiome composition (Tam et al. 2018; Willis et al. 2022). The α‐diversity of our study population is consistent with that of another study examining the oral microbiome in patients with MetS, reinforcing the broader applicability of our results (Tam et al. 2018).

Our findings indicate that modest clinical improvements in gingival inflammatory parameters, that is, BOP and GCF volume, can occur in patients with MetS undergoing a dietary intervention without adjunctive periodontal therapy. This observation aligns with previous studies on high‐fibre or otherwise modified diets, which similarly resulted in reductions in gingival inflammation and bleeding indices among patients with systemic conditions (Holmer et al. 2018; Jenzsch et al. 2009; Kondo et al. 2014). Notably, these interventions did not include subgingival instrumentation or intensified oral hygiene measures, yet yielded measurable local periodontal benefits. These data collectively suggest that targeted nutritional interventions may have a supportive role in modulating periodontal parameters, particularly in metabolically compromised populations.

Significant improvements in BOP, GBI and GCF volume post fasting align also with previous findings from the already published sub‐cohort of the female patients (Pappe et al. 2021). However, PI did not change in the original female cohort. In the present cohort, more patients were included, leading to a significant reduction in PI. The significant plaque reduction in this cohort may be due to fasting‐induced systemic modulation, lowering periodontal inflammation and bacterial growth substrates in the crevicular fluid (Hajishengallis et al. 2023).

On the contrary, also optimal oral hygiene/controlling the supragingival plaque levels results in significant reductions in pathogenic bacterial species and improvements in clinical measures such as probing depth and clinical attachment level (Gomes et al. 2008; Haffajee et al. 2003; Ximénez‐Fyvie et al. 2000). In the current study cohort, however, the observed stability of the supra‐ and sub‐gingival microbiome suggests that the reduction in plaque quantity has minimal direct impact on microbiota composition. It remains unclear whether the reduction in plaque directly mitigated inflammation or whether decreased inflammation facilitated lower plaque accumulation. It is plausible that these processes reinforce each other (Hämmerle et al. 1991; Pucher et al. 1997; Reiniger et al. 2021).

α‐Diversity and β‐diversity and dysbiosis measures did not show significant changes post fasting; only a trend towards reduced diversity after the 4–5‐month follow‐up was noted. These results are consistent with other studies following short‐term dietary interventions (Baumgartner et al. 2009; Woelber et al. 2019) and different fasting regimes (Lira‐Junior et al. 2024, Reynolds et al. 2009). For instance, an animal model in non‐human primates under 30% long‐term calorie restriction showed no effect on certain subgingival periodontal pathogens when compared to control animals. However, periodontal disease was more often diagnosed in animals under the *ad libitum* control diet (Reynolds et al. 2009). In a recent cohort study on intermittent fasting, no changes in subgingival diversity measures after 6 months were reported, but significant reductions in BOP and PPD were noted. When analysing only the ‘good responders’ who reduced ≥ 60% of their 4–5‐mm PPD, changes in several taxa were observed (Lira‐Junior et al. 2024). This change might also have been influenced by the pocket reduction and the environmental changes that followed (Shi et al. 2018). The lack of significant diversity changes suggests that the overall microbial community structure remained relatively stable, even though specific bacterial genera were affected. Thus, the observed anti‐inflammatory effect could be induced by fasting, which subsequently led to significant reductions in metabolic parameters such as FGLU and BMI.

Several genera showed consistent changes in relative abundance post fasting. In supra‐ and sub‐gingival samples, the genus Atopobium and genus *Mitsuokella* showed significant decreases, while *Eikenella* and Peptostreptococcaceae [XI][G‐7] showed significant increases.

*Mitsuokella*, typically isolated from dental root canals, decreased in sub‐ and supragingival samples. This genus is not commonly associated with the oral environment but was found in root canals, suggesting that its presence could be influenced by changes post fasting (Flynn et al. 1994; Haapasalo et al. 1986). The presence and behaviour of *Mitsuokella* may indicate its potential role in the altered environment due to fasting.

*Atopobium*, a facultative anaerobic genus that can occur in the oral environment, significantly decreased in sub‐ and supragingival samples. Notably, *Atopobium parvulum* contains a gene encoding a protein similar to bacterial tannases, which interacts with food tannins during oral processing. Although this enzyme has low specific activity and cannot hydrolyse complex tannins like tannic acid, its presence indicates that *Atopobium* might play a role in the breaking down of simpler tannins (Jiménez et al. 2014). However, species‐level analyses did not support genus‐level findings of decreased levels of *Atopobium*.

*Eikenella*, which increased in both subgingival and supragingival samples, has been associated with various forms of periodontitis, particularly in younger individuals. Its prevalence is known to decrease with age in healthy individuals but remains relatively constant in those with periodontitis (Suda et al. 2002). The observed increase in *Eikenella* at the genus level should also be interpreted with caution, given that *Eikenella corrodens* , as found in the species level analysis of sub‐ (Suppl. Figure S3), and supra‐ (Suppl. Figure S4) gingival samples, is considered to be associated with periodontitis in some contexts but can also be detected in healthy sites (Feres et al. 2021).

*Peptostreptococcaceae* [XI][G‐7], an uncultivable oral genus, was found to be associated with sucrose intake in another study (Esberg et al. 2020). Its increase in both subgingival and supragingival samples post fasting could indicate dietary shifts influencing its abundance. From a periodontal standpoint, the significance of such alterations must be carefully evaluated. Specifically, *Peptostreptococcaceae* [XI][G‐7] is often associated with periodontitis rather than health (Chen et al. 2022; Colombo et al. 2021; Pérez‐Chaparro et al. 2014).

*Lachnospiraceae* [G‐3], which showed significant increases only in subgingival samples, belong to the core of gut microbiota, colonising the intestinal lumen from birth and increasing in terms of species richness and relative abundances during the host's life (Vacca et al. 2020). Members of *Lachnospiraceae* are among the main producers of short‐chain fatty acids, which play crucial roles in maintaining gut health. However, different taxa of *Lachnospiraceae* are also associated with various intra‐ and extra‐intestinal diseases, and their impact on host physiology is often inconsistent across different studies. The increase in *Lachnospiraceae* [G‐3] post fasting could suggest complex interactions between dietary changes, oral health and systemic immune responses. These changes might reflect a shift in metabolic activities or a response to altered nutrient availability due to fasting.

Although this study provides valuable insights, certain aspects warrant further exploration. The absence of a non‐fasting control group influences the interpretation of the results because we cannot distinguish effects resulting solely from fasting or other effects from the stay at the fasting clinic. Interactions with plaque scores were not fully explored, which should be better controlled in future studies. The use of short‐read 16S rRNA gene sequencing offers important information but limits species‐level resolution; employing methods with deeper taxonomic resolution could enhance understanding. Additionally, assessing microbiome function through metagenomics or metatranscriptomics, as well as evaluating host immune responses, would provide a more comprehensive view of the underlying mechanisms. The focus on site‐specific plaque samples suggests that including a broader range of sample types might yield additional insights. The reduced sample size, partly due to loss to follow‐up, affected the statistical power to detect changes after 4–5 months. It is important to note that the present study was designed primarily as an exploratory analysis rather than a definitive clinical trial. Our findings, therefore, should be interpreted as hypothesis‐generating observations that warrant further examination. Future research, ideally involving larger cohorts, an a priori power calculation and randomised controls, is needed to confirm these preliminary results and to delineate more precisely the mechanisms by which prolonged fasting may influence periodontal parameters and the oral microbiome. Further, future studies should ideally include multiple pre‐treatment sampling timepoints to better capture intra‐individual variability in the microbiome. Exploring different fasting durations and regimes, and examining interactions with the gut microbiome, could also be valuable directions for further investigation.

## 5 Conclusion

Prolonged fasting leads to a set of unique changes within the oral microbiome that are not all clear markers of periodontal health or disease but modestly improves inflammatory periodontal parameters in patients with MetS. However, our single‐arm design and limited sample size underscore that these findings remain exploratory, warranting larger controlled trials to confirm their broader clinical relevance.

## Author Contributions

C.L.P. was responsible for the study coordination, study design, examinations and writing the manuscript. S.K.B. was responsible for data analysis and critically reviewing the manuscript. K.P. conducted microbiome sequencing and critically reviewed the paper. B.E., A.M. and N.S. contributed to critically reviewing the paper. H.D. contributed to study conduction and critically reviewed the paper. D.H. was responsible for funding, data analysis and interpretation as well as writing the manuscript. All authors granted their approval for the final version of the paper and reached a consensus on all aspects of the work.

## Conflicts of Interest

The authors declare no conflicts of interest.

## Supporting information


**Figure S1.** Boxplots of α‐diversity measures (richness, Shannon, Simpson) for supragingival (supra) and subgingival (sub) and subgingival dysbiosis index (SMDI) for subgingival samples at baseline (T1) and post fasting (T2). Boxes are coloured by timepoint (blue for T1, orange for T2) and patterned by site (plain for supragingival, cross‐hatched for subgingival). The diversity measures are presented as median and interquartile ranges, with no differences detected between T1 and T2 controlled for age, differences in fasting duration and sequencing depth.

**Figure S2.** Principal coordinates analysis (PCoA) plots of Bray–Curtis dissimilarity for supragingival (A, cross‐hatched) and subgingival (B, plain) samples at baseline (T1) and post fasting (T2). Points are coloured by timepoint (blue for T1, orange for T2). The ellipses represent 95% confidence intervals. The separation along PC1 and PC2 indicates changes in microbial community structure. Arrows show the main drivers of variation as assessed by distance‐based redundancy analysis (dbRDA) controlling for age, sex, timepoint, smoking and fasting duration.

**Figure S3.** Differential abundance summary for subgingival, showing the relative abundance (log_10_ scale), prevalence and log_2_‐fold change of species‐level taxa significantly different between baseline (blue) and post fasting (orange). Species are marked green when they show significantly different abundances in the respective models (ANCOM, LinDA and Wilcoxon).

**Figure S4.** Differential abundance summary for supragingival samples, showing the relative abundance (log_10_ scale), prevalence, and log_2_‐fold change of species‐level taxa significantly different between baseline (blue) and post fasting (orange). Species are marked green when they show significantly different abundances in the respective models (ANCOM, LinDA and Wilcoxon).

**Table S1.** De‐noised sequences and taxonomic classifications down to the species level for the human oral microbiome database (HOMD) and the Silva Database (SILVA).

## Data Availability

The data that support the findings of this study are openly available in European Nucleotide Archive at http://www.ebi.ac.uk/ena/, reference number PRJEB89223.
