# Supplementary figures and images for: Impact of Prolonged Fasting on the Oral Microbiome in Patients With Metabolic Syndrome: An Exploratory Secondary Analysis
Source: J Clin Periodontol. 2025 May 12;52(8):1125–35. doi: 10.1111/jcpe.14171 (PMC12259404; doi:10.1111/jcpe.14171)

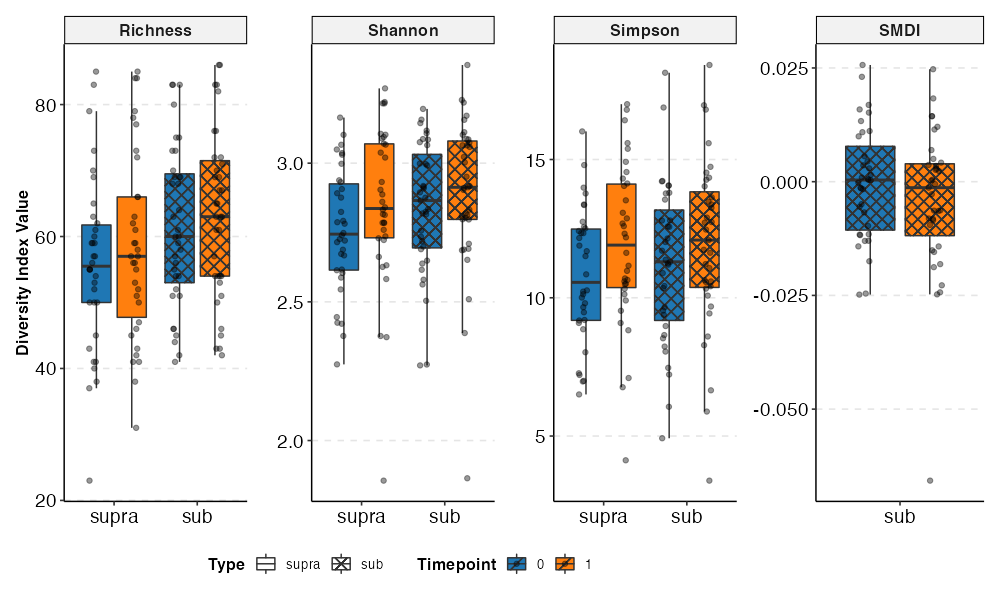

Supplement: Supplementary file 1 — Figure S1. Boxplots of α‐diversity measures (richness, Shannon, Simpson) for supragingival (supra) and subgingival (sub) and subgingival dysbiosis index (SMDI) for subgingival samples at baseline (T1) and post fasting (T2). Boxes are coloured by timepoint (blue for T1, orange for T2) and patterned by site (plain for supragingival, cross‐hatched for subgingival). The diversity measures are presented as median and interquartile ranges, with no differences detected between T1 and T2 controlled for age, differences in fasting duration and sequencing depth. [file JCPE-52-1125-s002.tiff]

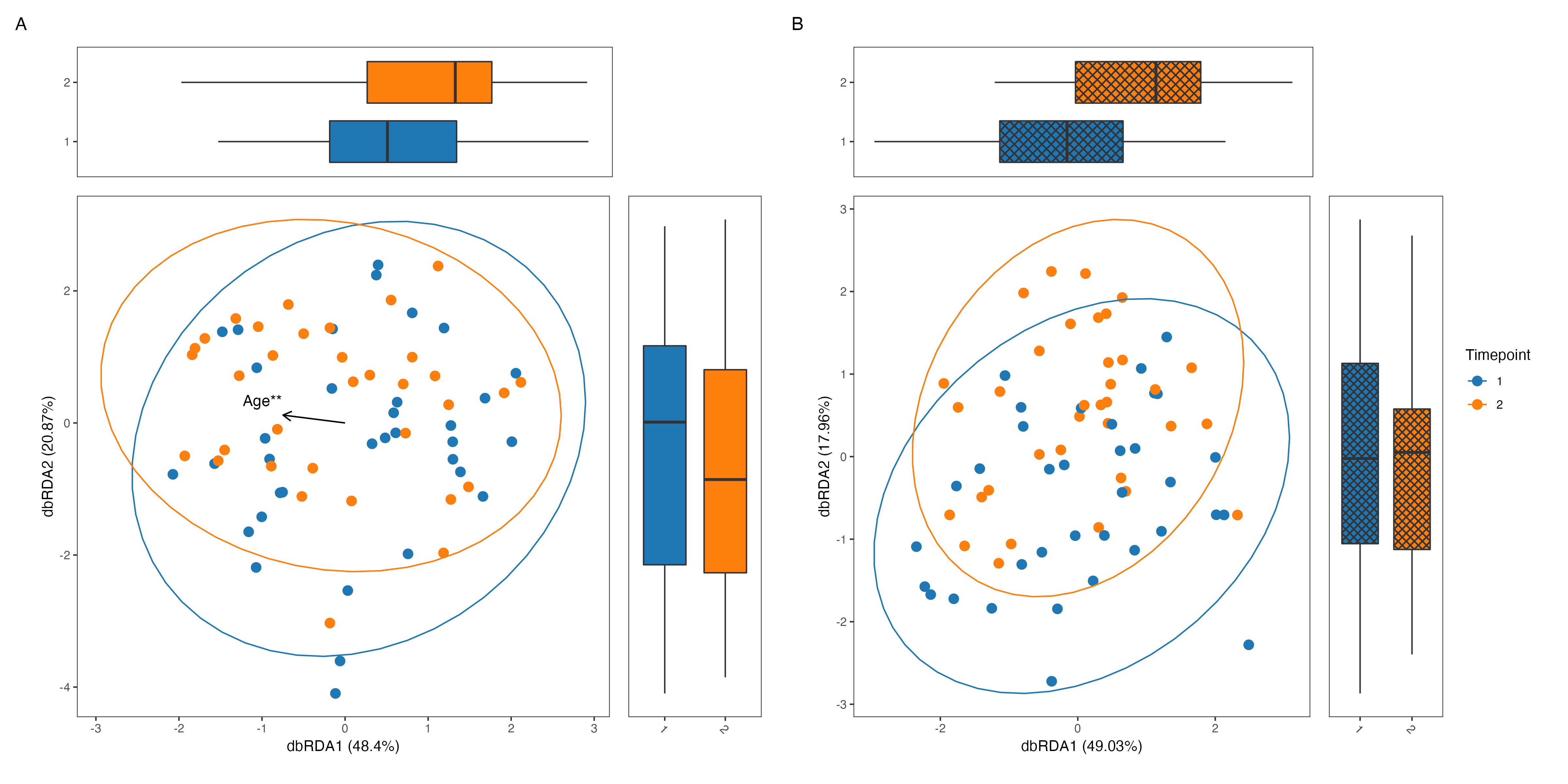

Supplement: Supplementary file 2 — Figure S2. Principal coordinates analysis (PCoA) plots of Bray–Curtis dissimilarity for supragingival (A, cross‐hatched) and subgingival (B, plain) samples at baseline (T1) and post fasting (T2). Points are coloured by timepoint (blue for T1, orange for T2). The ellipses represent 95% confidence intervals. The separation along PC1 and PC2 indicates changes in microbial community structure. Arrows show the main drivers of variation as assessed by distance‐based redundancy analysis (dbRDA) controlling for age, sex, timepoint, smoking and fasting duration. [file JCPE-52-1125-s005.tiff]

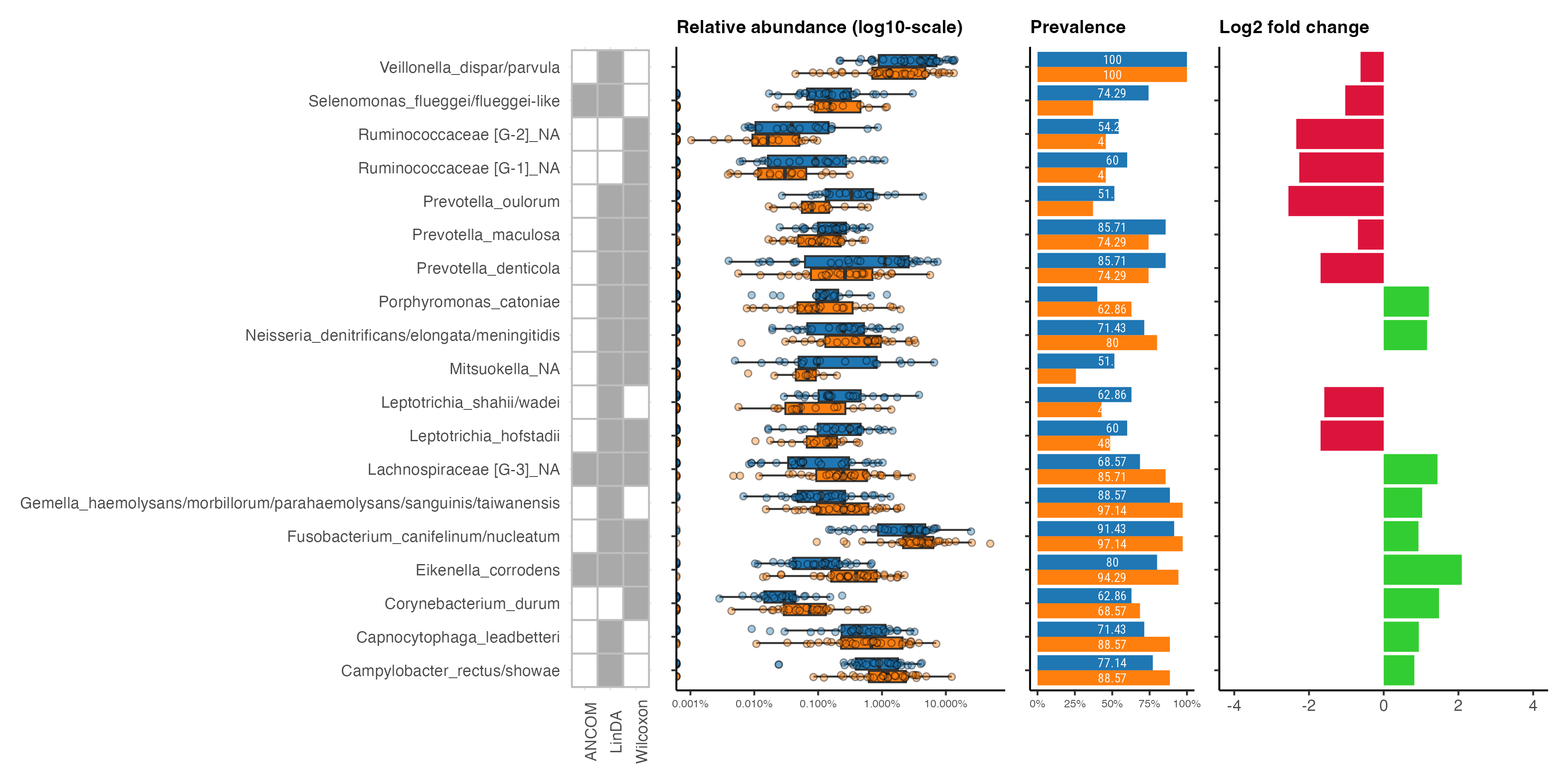

Supplement: Supplementary file 3 — Figure S3. Differential abundance summary for subgingival, showing the relative abundance (log10 scale), prevalence and log2‐fold change of species‐level taxa significantly different between baseline (blue) and post fasting (orange). Species are marked green when they show significantly different abundances in the respective models (ANCOM, LinDA and Wilcoxon). [file JCPE-52-1125-s004.tiff]

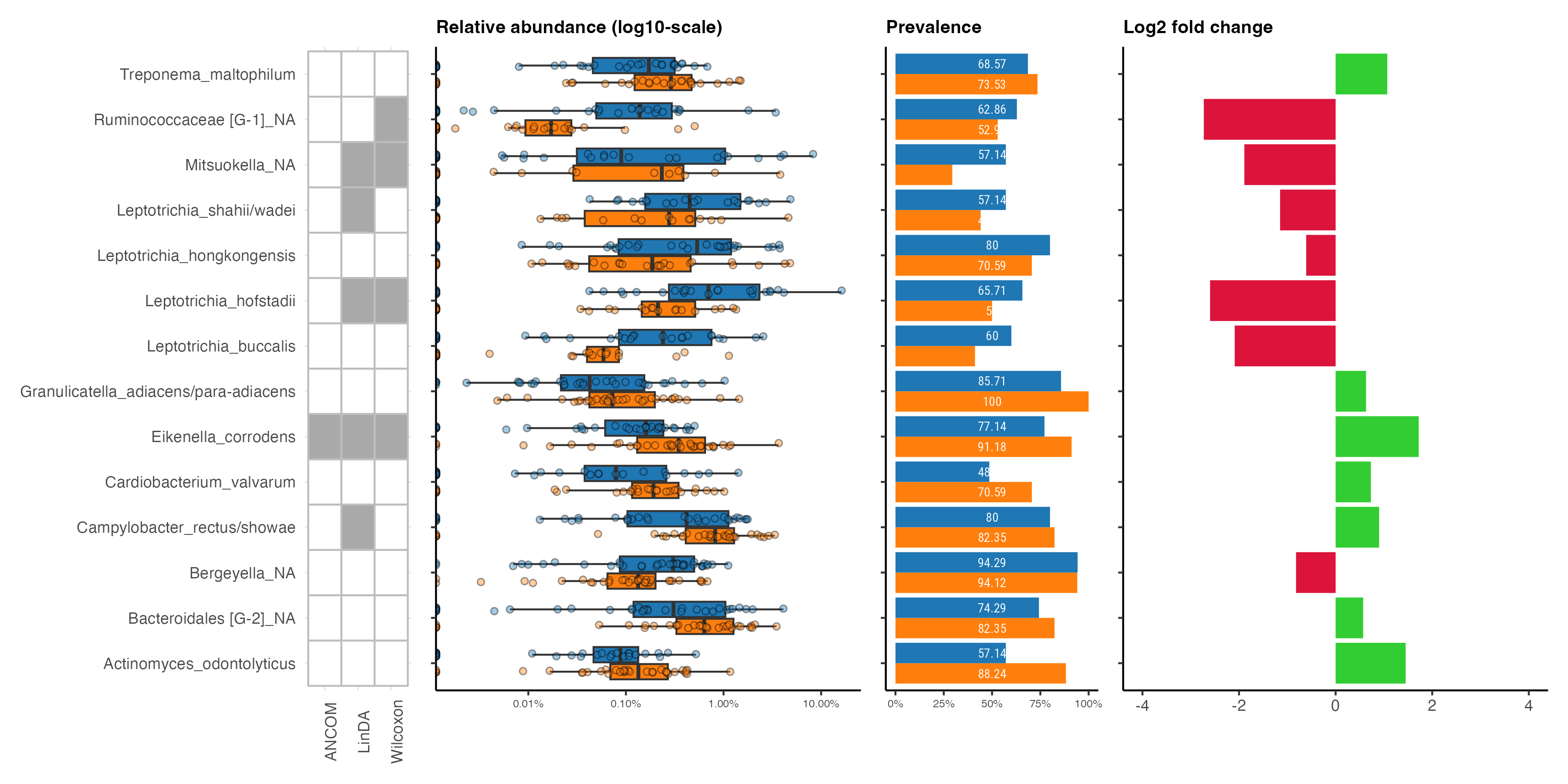

Supplement: Supplementary file 4 — Figure S4. Differential abundance summary for supragingival samples, showing the relative abundance (log10 scale), prevalence, and log2‐fold change of species‐level taxa significantly different between baseline (blue) and post fasting (orange). Species are marked green when they show significantly different abundances in the respective models (ANCOM, LinDA and Wilcoxon). [file JCPE-52-1125-s001.tiff]
